# Chromium and lead levels and alteration in DDPH inhibition in patients with breast cancer undergoing chemotherapy 

**DOI:** 10.22088/cjim.14.3.533

**Published:** 2023

**Authors:** Fatemeh Pakmanesh, Soleiman Mahjoub, Nahid Neamati, Daryush Moslemi

**Affiliations:** 1 Student Research Committee, Babol University of Medical Sciences, Babol, Iran.; 2 Cellular and Molecular Biology Research Center, Health Research Institute, Babol University of Medical Sciences, Babol, Iran; 3 Department of Clinical Biochemistry, School of Medicine, Babol University of Medical Sciences, Babol, Iran; 4 Cancer Research Center, Health Research Institute, Babol University of Medical Sciences, Babol, Iran

**Keywords:** Adriamycin, Cytoxan, DPPH inhibition, Chromium, Lead, Breast cancer, Chemotherapy

## Abstract

**Background:**

Recently the carcinogenic and toxic effects of some heavy metals such as chromium (Cr), and lead (Pb) through the mechanism of oxidative stress have been reported. Due to the various consequences of chemotherapeutic treatments on body hemostasis, the present study aimed to evaluate the effect of Adriamycin 60 mg/m^2^ and Cytoxan 600 mg/m^2^ (AC) chemotherapy on the serum levels of Cr, Pb, and the percent α-diphenyl-β-picrylhydrazyl (DPPH) inhibition.

**Methods:**

This study was performed on 50 patients with breast cancer at two separate sampling times, the first at the initiation of chemotherapy and the last at the end of three courses of the AC chemotherapy treatment. Serum levels of Cr and Pb were measured using atomic absorption spectrophotometry. The percent DPPH inhibition (% I) and also the effect of age and stage of the disease on the mentioned variables were evaluated. Statistical comparison of the obtained results before and after chemotherapy was performed using paired sample t-test. Intra-group evaluation of age and disease stages was done using an independent sample t-test.

**Results:**

A significant decrease was observed in the percent DPPH inhibition after 3 courses of chemotherapy (p<0.001). Cr and also Pb were significantly higher in patients with breast cancer after AC chemotherapy (p<0.001).

**Conclusion:**

According to the results, AC chemotherapy in patients with breast cancer is associated with higher levels of Cr and Pb, which can eventually lead to worsened oxidative stress status in affected patients. However, it seems that these changes do not necessarily depend on age and the stage of the disease.

Breast cancer, one of the most common neoplasms around the world, is accounted for the causes of death, especially in women aged 40-50 years old, in most developing countries (1). It is not unique to women and men also may be affected (2). The uncontrolled spontaneous proliferation of malignant cells is not morphologically the same as normal tissues. Cancerous tissues are often undifferentiated and have diverse characteristics than normal ones. They have different biological characteristics and cannot protect themselves against oxidative DNA damage (3).^. ^Usually, cancerous cells can immigrate to the other parts of the body and substitute for the normal tissue, i.e. metastatic properties of cancer cells, which may occur when cancer cells enter the body's circulatory system (4). Among all the factors affecting the growth and progression of metastatic cancer, oxidative stress and especially the weakness of body oxidative defense, i.e. disturbances of antioxidant enzymes and trace elements metabolism, play an important role in cancer development by damaging DNA and interfering with intracellular pathways (5-7). 

It is well characterized by macromolecular damage to lipids, nucleic acids, and also protein components in our body system (8,9). Interestingly, it is said that some trace elements can exert their oncogenic or protective effects by interfering with the formation of reactive oxygen species (ROS). These effects are identified as oxidative damage factors on biological macromolecules and DNA. However, their exact role in the development of cancer is not well understood (10,11). 

A large number of previous studies have shown a close relation between heavy metals such as chromium (Cr), lead (Pb), copper (Cu), arsenic (As), cadmium (Cd), and nickel (Ni) with the development of breast cancer (12-15). These compounds can be present in various sources including free ions, solid metal compounds, or solutions containing carcinogenic heavy metals (16). Some of the reports have focused on the relationship between these metals and the risk of breast cancer (17). In general, the carcinogenic effects and toxicity of these metals are based on three main mechanisms, including oxidative stress, altering the structure of DNA and inducing impaired cellular signaling pathways (16). 

Atukeren et al. reported decreased levels of total antioxidant capacity (TAC) in patients with breast cancer compared to the healthy control group. In addition, the same result was observed after chemotherapy (18). In a study by Saleh et al., the serum levels of copper (Cu), zinc (Zn), selenium (Se), and cadmium (Cd) in 50 patients with breast cancer were compared against 150 healthy individuals. In patients with breast cancer, serum Cu, Z,n and Se levels were significantly lower than in the control group. However, a significantly higher mean value of cadmium was observed (19). Ragab et al. measured the serum levels of some trace elements (including Pb, Cd, Cr, Fe, Ni) and TAC in 120 patients (100 women with breast cancer and 20 with benign breast disease). As a result of their study, significant increases in the concentration of measured elements in the patients compared to the control group were observed (P<0.001). Moreover, the levels of TAC were significantly lower in patients with breast cancer compared with the control group (20,21). 

Higher levels of blood or tissue Cr were reported in patients with bladder (22), head and neck carcinoma (23), and gastrointestinal malignancies (24). A proposed adverse effect of Cr is attributed to interaction with intracellular DNA which causes DNA damage and its mutagenic complications (25). There are a few inconsistent studies on the effects of chemotherapy drugs such as Doxorubicin (Adriamycin-AC) on heavy metals in patients with breast cancer. This study aimed to investigate the effect of AC chemotherapy on variations of the serum Cr and Pb levels in this treatment strategy with regard to the age and stage of this disease.

## Methods

**Study design: **The present study was approved by the Ethics Committee of Babol University of Medical Sciences (IR.MUBABOL.HIR.REC.1395.21) and written consent was obtained from all participants in the study. 2- Participants. The study group consisted of 50 patients with breast cancer, referred to Shahid Rajai Hospital (Babolsar, Iran) and Ayatollah Rouhani Hospital (Babol, Iran), who were diagnosed according to pathological examination by a specialist physician. 

Participants’ inclusion criteria included ages between 31-74 years old who were prescribed chemotherapy followed by surgery. Moreover, according to the TNM score, all patients with ductal carcinoma were scored in stages II and III of the disease. Exclusion criteria included people with diabetes, inflammatory diseases, collagen, and vascular diseases as well as consumers of vitamin and antioxidant supplements. Patients who had a history of chemotherapy were also excluded. Blood sampling was done in two steps (first sampling was done before chemotherapy and the second was performed 3 weeks after the chemotherapy, the last sampling was performed after three chemotherapy courses.) Chemotherapy drugs included Adriamycin 60 mg/m2 and Cytoxan 600 mg/m2, which were injected intravenously and once every 3 weeks. The sera of the subjects were collected in Eppendorf conical tubes and stored at -80° C until analysis. 

**Evaluation of Serum Cr and Pb: **Serum levels of Pb and Cr were measured by Atomic Absorption Spectroscopy (AAS) equipped with a graphite furnace. 

**DPPH free radical scavenging** **assay**: The ability of free radical scavenging activity as the antioxidant index was evaluated by 2,2-diphenyl-1-picrylhydrazyl (DPPH) assay. DPPH assay is a reliable method for the evaluation of antioxidant activity in biological fluids (26). In this method, the antioxidant activity can be defined by the amount of reduced DPPH radicals. Antioxidant activity converts the DPPH radical to the reduced form and the color of the solution changes from purple to yellow. Decreased absorbance at 517 nm is a sign of higher reduction of DPPH and greater antioxidant activity.

**Statistical methods: **Statistical analysis was performed by SPSS software (Version 18). Results have been reported as mean ± SD or number (%). To evaluate the effect of chemotherapy on the general level of indices before and after chemotherapy, paired t-test and independent t-test were used. Finally, a p-value less than 0.05 was considered significant.

## Results

This study was a case-control study on serum samples of 50 patients (31-74 years old) with breast cancer before and after chemotherapy. The clinical and demographic characteristics of the patients are represented in table 1. All pre-chemotherapy information is present in table 1. TNM scoring was defined according to tumor size (T), lymph node involvement (N), and metastasis to other tissues (M). In addition, hormone receptors (Her-2, PR, ER), as well as the stage of the disease, were also studied. 

**Table 1:** Basic and clinical characteristics of the patients

**Characteristics**	**Number**	**Percent**
Age (median) <48 years >48 years	22 28	44 46
Familial background of cancer Absent Present	41 9	82 18
Cancer site Right breast Left breast	23 27	46 54
Type of surgery Lumpectomy Mastectomy	9 41	18 82
Cancer stage Early Advanced	32 18	64 36
Cancer Grade G1 G2 G3	12 35 3	24 70 6
TNM score Tumor size T1 T2 T3 T4	7 29 13 1	14 58 26 2
Lymph node involvement N0 N1 N2 N3	14 23 7 6	28 46 14 12
Metastasis M0 M1 Mx	34 8 8	68 16 16
Hormone receptors Estrogen receptor ER-positive ER-negative	34 16	68 32
Progesterone receptor PR-positive PR-negative	33 17	66 34
HER-2 status HER-2 positive HER-2 negative	18 32	36 64

ER; Estrogen Receptor, PR; Progesterone Receptor, HER-2; Human epidermal receptore-2, Early stage: stage I, II, Advanced stage: stage III, IV

The effect of chemotherapy on serum levels of Pb, Cr and Free radical scavenging activity percentage (% I) represent their variations before and after chemotherapy which are presented in table 2. All data are present as mean ± SD. According to table 2, in the present study, a significant reduction was observed in their percentage of free radical scavenging activity (P<0.001). Serum Cr and Pb increased significantly higher (P<0.001 and 0.046, respectively) after chemotherapy. In addition, there was no significant change in BMI index. Taking into account the stage of the breast cancer, serum levels of Cr, Pb, and I% changes were compared before and after chemotherapy in early and advanced stages of patients with breast cancer (table 3). 

**Table 2:** Serum Cr, Pb, and DPPH changes before and after chemotherapy

**Variable**	**Before**	**After**	**P-value**
**DPPH** ** (I %)**	11.3±1.8	7.5±1.8	<0.001
** Cr (μg/L)**	3±1.3	4.7±2.5	<0.001
**Pb (μg/L)**	4.1±1.7	4.8±2.5	0.046
** BMI (kg/m²)**	27.7±3.8	27.8±3.7	0.48

DPPH: α-diphenyl-β-picrylhydrazyl, Cr: Chromium, Pb: Lead, BMI: Body mass index

**Table 3:** Comparison between serum Cr, Pb, and DPPH changes before and after chemotherapy in early and advanced stages of the breast cancer

**Variable**	**Cancer staging**	**Before**	**After**	**P-value**
**DPPH (I %)**	Early	11.7±1.6	7.9±1.5	<0.001
	Advanced	10.7±2.1	6.9±2	<0.001
	P-value	0.065	0.061	--
**Cr (μg/L)**	Early	3.3±1.3	4.8±2.6	0.005
	Advanced	2.5±1.3	4.6±2.5	0.007
	P-value	0.061	0.741	--
**Pb (μg/L)**	Early	4.1±1.7	4.9±2.6	0.076
	Advanced	4.1±1.9	4.6±2.5	0.373
	P-value	0.952	0.741	--
**BMI (kg/m²)**	Early	27±3.6	27.1±3.7	0.456
	Advanced	29±3.8	29.1±3.6	0.818
	P-value	0.075	0.074	--

DPPH: α-diphenyl-β-picrylhydrazyl, Cr: Chromium, Pb: Lead, BMI: Body mass index

As it has been described before, patients in stages I and II of cancer were considered as early and the others as advanced stages. With regard to the stage of cancer, no statistical difference was observed between the early and advanced stages. However, after chemotherapy, a significant decrease in I % was clear in each stage. Serum Cr was significantly higher after chemotherapy. 

But such a result was not observed in serum Pb changes (table 4).

 According to table 4, the percentage of I% was higher in patients more than 48 years old. Regardless of age classification, after chemotherapy, a significant reduction in I% and an increase in Cr was observed in each age classification. 

**Table 4:** Comparison between serum Cr, Pb, and DPPH changes before and after chemotherapy with considering the age of the patients with breast cancer

**Variable**	**Cancer staging**	**Before**	**After**	**P-value**
**DPPH (I %)**	< 48 years	10.8±1.8	7 ± 1.7	<0.001
	> 48 years	12±1.7	8.2±1.6	<0.001
	P value	0.028	0.020	--
**Cr (μg/L)**	< 48 years	3±1.5	4.8±2.6	0.002
	> 48 years	3±1.1	4.6±2.6	0.017
	P value	0.984	0.798	--
**Pb (μg/L)**	< 48 years	4.2±1.9	4.9±2.6	0.132
	> 48 years	4±1.6	4.7±2.4	0.208
	P value	0.749	0.798	--
**BM** **I** **(kg/m²)**	< 48 years	28.2±3.8	28.3±3.7	0.581
	> 48 years	27.1±3.8	27.2±3.8	0.680
	P value	0.312	0.298	--

DPPH: α-diphenyl-β-picrylhydrazyl, Cr: Chromium, Pb: Lead, BMI: Body mass index

## Discussion

In the present study, less than 20% of breast cancer patients had a familial background. There was no significant difference in left and right breast disease. 70% of our patients had grade 2 (G2) and had M0 status in terms of metastasis. About 70% of patients were ER & PR-positive, but more than 60% of them were HER-2 negative. Also, more than 60% of patients were diagnosed in the early stage of breast cancer, which compared to some studies, indicates earlier diagnosis. According to our findings, the inhibition of the DPPH percentile (I%) significantly decreased after treatment with the AC chemotherapy compared with pre-treatment in patients with breast cancer. Chemotherapy is an optional treatment that may be prescribed periodically for those patients without estrogen and progesterone receptor. Chemotherapy drugs can cause various complications in the metabolism and general body status. It can result in increased oxidative stress and reduced antioxidant capacity. 

To achieve a better outcome, a combination of some chemical drugs which usually involve more than one type of drug may be prescribed. It is noteworthy that the dosage of the drug and the duration of treatment can vary according to the stage of the disease. There are some reports on the relation between heavy metals and the risk of diseases (15, 27). For example in breast cancer, metal compounds can interact with estrogen function (17). In our research, increased serum levels of Cr and Pb were observed after chemotherapy compared with pre-treatment in patients with breast cancer who were treated with Adriamycin and Cytoxan chemotherapy. However, by age or stage of cancer classifications, there was not a big discrepancy in results. The effect of the drugs on the serum levels of Cr and Pb as trace elements, as well as the antioxidant index (% I) were evaluated in patients with breast cancer.

In a study by Atukeren et al. including 30 patients before and after the first and second courses of chemotherapy, there was a significant reduction in total antioxidant capacity of patients with breast cancer compared with healthy individuals and after chemotherapy (18). In line with their research, the I% in our study had a significant reduction after chemotherapy. It is noted that in the present study, our sampling was done with more participants and in the third period of chemotherapy. Moreover, we also evaluated the trace elements’ status in included participants. In the study by Saleh et al., 50 patients with breast cancer had significantly lower serum levels of Cu, Zn, and Se compared with the control group, and the mean cadmium levels as heavy metal were higher than the control group (19). We measured other heavy metals i.e. Cr and Pb before and after chemotherapy, which showed a significant increase after the AC chemotherapy. In the study of Ragab et al. which included 120 patients, a significant increase in the concentration of trace elements, especially chromium and lead, and a decrease in total antioxidant capacity levels were observed (20). The same results were also observed in our study. In addition, our study was done before and after chemotherapy, and the ability of free radical scavenging activity as the antioxidant index was measured using the DPPH method.

In this study, there was a significant increase in the level of trace elements including chromium and lead (P <0.05), but the status of the indices studied in different age groups and stages of the disease was not significant. Also, the BMI of the patients who were identified in the advanced stages of the disease was higher compared to the patients in the early stages, but this difference was not significant. Atukeren et al. examined total antioxidant capacity (TAC) in 30 patients with breast cancer at pre-treatment and after the first and second cycle of chemotherapy compared with 20 healthy subjects as controls. They reported a significant decrease in TAC in patients compared to the control group and also the same result was observed after chemotherapy (18). Our findings indicate that the AC chemotherapy regimen in patients with breast cancer causes a significant change in the body's oxidant / antioxidant system and significantly changes the level of trace elements. Of note, their changes do not necessarily depend on the patient's age and stage of cancer. Studying human samples always has abundant limitations and challenges, for example, it remains doubtful whether the subjects studied were in equal condition or not. In this research, we tried to ensure that patients were treated in the same way in terms of nutrition and treatment as well as age and stage rankings. The present study examined the effect of AC chemotherapy after three periods of breast cancer. In the future, long-term studies on metabolism changes in different chemotherapy regimens and patient response to recurrence or improvement of the tumor are suggested. According to our findings, AC chemotherapy leads to a decreased antioxidant index and an increase in serum levels of Cr and Pb, which can eventually lead to worsened oxidative stress status in patients with breast cancer. Antioxidant-rich foods and antioxidant supplements are recommended to combat oxidative stress after chemotherapy courses. 
